# Long-Term Survival of Metachronous Isolated Adrenal Metastasis in Luminal Breast Cancer: A Case Report and Literature Review

**DOI:** 10.7759/cureus.78142

**Published:** 2025-01-28

**Authors:** Griet Verboven, Manon T Huizing, Maarten Weijer, Dirk Ysebaert, Ali Ramadhan, Tim Wyngaert, Glenn Broeckx, Wiebren A Tjalma

**Affiliations:** 1 Faculty of Medicine and Health Sciences, University of Antwerp, Antwerp, BEL; 2 Department of Obstetrics and Gynecology, Antwerp University Hospital, Edegem, BEL; 3 Department of Medical Oncology, Antwerp University Hospital, Edegem, BEL; 4 Department of Radiology, Antwerp University Hospital, Edegem, BEL; 5 Department of Hepatobiliary, Endocrine and Transplantation Surgery, Antwerp University Hospital, Edegem, BEL; 6 Department of Pathology, Antwerp University Hospital, Edegem, BEL; 7 Department of Nuclear Medicine, Antwerp University Hospital, Edegem, BEL; 8 Department of Pathology, Gasthuiszusters van Antwerpen (GZA) Ziekenhuis Netwerk Antwerpen (ZNA) Hospitals, Antwerp, BEL

**Keywords:** breast cancer metastasis, long term survivor, luminal-like breast cancer, minimally invasive adrenalectomy, oligo metastatic disease, tumor dormancy

## Abstract

Metachronous metastasis occurs many years later in cases of hormone-positive, human epidermal growth factor receptor 2 (HER2)-negative breast cancer, with the most common sites being the lymph nodes, bones, liver, lungs, and brain. The late recurrence of estrogen receptor (ER)-positive breast cancer is attributed to prolonged adjuvant therapy and the high expression of dormancy-associated genes, allowing cancer cells to survive for decades without proliferating. It is a form of chronic breast cancer that remains asymptomatic, with no clinical signs of progression or recurrence. Estrogen receptor-negative breast cancers, on the other hand, have no long-term tumor dormancy due to their fast growth and low expression of dormancy-related genes. Adrenal gland metastasis, particularly as an oligometastatic presentation, is exceedingly rare, and optimal treatment strategies remain elusive.

In this report, we present a case demonstrating long-term survival after treatment of adrenal gland metastasis, accompanied by a comprehensive literature review. At the age of 48, our patient was diagnosed with invasive ductal carcinoma of the left breast. Treatment included breast-conserving surgery, radiotherapy, and adjuvant hormone therapy. Ten years later, she developed a solitary left adrenal metastasis. Treatment included laparoscopic adrenalectomy and a change in hormonal therapy. Our patient is currently still asymptomatic with no evidence of disease. Her overall survival of over 20 years is exceptional.

Resection of the adrenal metastasis combined with systemic hormonal therapy represents the recommended approach for this metachronous metastasis. In the contemporary context, antihormonal therapy in combination with CDK4/6 inhibitors should be considered. The present case underscores the necessity of establishing a lifelong (inter)national cancer registry to document rare recurrences systematically. Such a registry would provide insights into the prevalence of uncommon scenarios and offer invaluable data on proposed treatments, facilitating the development of uniform treatment strategies.

## Introduction

Breast cancer is the second most common disease in women worldwide and the fourth leading cause of cancer-related death. Recent data indicates 2,296,840 new diagnoses (age-standardized rate (ASR) 46.8) and 666,103 deaths (ASR 12.7) annually [1]. The two most common types of invasive breast cancer are breast carcinoma of not special type (NST), formerly known as ductal carcinoma, accounting for 70% to 75% of cases, and lobular carcinoma, which comprises 12% to 15% of cases. The remaining 18 types exhibit specific morphological features and are relatively rare, with individual frequencies ranging from 0.5% to 5% [2].

Breast cancer can be classified into four intrinsic subtypes: luminal A, luminal B, human epidermal growth factor receptor 2 (HER2)-positive, and basal-like. This classification can be done using multigene assays or indirectly through immunohistochemistry (IHC), based on the expression of hormone receptors (estrogen receptor (ER) and progesterone receptor (PR)), HER2 status, and Ki67 (a proliferation marker) [2]. The stage and subtype of breast cancer significantly influence recurrence and survival rates [2-4]. The cumulative incidence of late recurrence over 20 years in ER-positive tumors is nearly double that of ER-negative tumors (8%) [5]. For T1N0 cancers, the annual distant recurrence rate is approximately 1% from five to 20 years post-diagnosis, resulting in a cumulative distant recurrence risk of 13% [6].

Chronic cancer without signs or symptoms of progression or recurrence is the clinical translation for tumor dormancy or cancer cells in a quiescent state. Our case was and is an example of chronic breast cancer. In chronic breast cancer, progression or recurrence occurs when the balance between the tumor cells and the microenvironment is disturbed. It has been calculated that the annual recurrence rate after 10 years is about 1% for ER-positive tumors and less than 0.3% for ER-negative tumors [5,7]. Based on this theory, a so-called local recurrence after 10 years or more is therefore more likely to be a new primary disease than a true recurrence.

Dormancy differs significantly between ER-positive and ER-negative breast cancers, mainly due to hormone dependency and gene expression. Estrogen receptor-positive cancers often enter a dormant state during anti-hormonal therapy, as the reduced estrogen levels keep tumor cells inactive; however, stopping therapy can stimulate dormant cells, increasing the risk of recurrence [8,9]. In addition, ER-positive cancers have high expression of dormancy-related genes, which increases their resistance to cell death and allows them to survive for decades [10,11]. Conversely, ER-negative tumors generally have lower expression of dormancy-related gene expression, resulting in faster progression or response to treatment and fewer cases of late recurrence [12].

The most common metastatic sites are the lymph nodes, bone, liver, lung, and brain [13]. Adrenal metastasis is extremely rare in breast cancer, with an incidence of 0.25%, but is more commonly associated with lung cancers (35.4%), gastric cancers (14.3%), esophagus cancers (12.1%), liver cancers (10.7%), intestinal cancers, hematopoietic neoplasms (lymphomas/leukemias), renal cell carcinoma, and melanoma [14,15]. Adrenal metastasis is noteworthy because it can disrupt the production of hormones such as cortisol, aldosterone, and adrenaline. Clinically, this disruption can lead to symptoms like fatigue, weight loss, low blood pressure, and electrolyte imbalances.

Metachronous metastasis is defined as a distant recurrence three months or more after the treatment of the primary tumor, whereas synchronous metastasis occurs at the same time as the primary tumor. When adrenal metastasis occurs in breast cancer, it is usually not in a metachronous oligometastatic form but as part of a broader synchronous metastatic presentation. The rarity of such cases complicates the development of optimal treatment strategies. This report describes a case of isolated metachronous adrenal metastasis in a patient with a history of NST breast cancer and presents a literature review on isolated metachronous adrenal metastasis in breast cancer, focusing on management recommendations.

## Case presentation

At the age of 48, our patient (G2P2A0) was diagnosed with a pT1cpN0M0 invasive ductal carcinoma of the left breast. The tumor size was 18 mm and poorly differentiated (grade 3). The ER was positive (80%) while the PR was weakly positive (10%). Human epidermal growth factor receptor 2 was negative by IHC and fluorescence in situ hybridization (FISH). The clinical course of the patient is outlined in Table 1.

**Table 1:** Timeline of our patient

Age (years)	Incident	Treatment
48	Invasive ductal cancer of the left breast (pT1cpN0M0)	Breast conservative surgery | Adjuvant radiotherapy | Hormonal therapy: Tamoxifen and goserelin
52	A recurrence in a right supraclavicular node and possibly a mediastinal node	Radiotherapy on internal mammary nodes and supraclavicular node stations | Hormonal therapy: Switch to exemestane and goserelin | Goserelin was discontinued after bilateral ovariectomy
56	A recurrence in the contralateral right axilla (solitary node)	Excision of nodule | Adjuvant chemotherapy: 4x AC and 4 x T | Radiotherapy | Hormonal therapy: Switch to letrozole
58	A solitary left adrenal metastasis	Laparoscopic adrenalectomy | Hormonal therapy: Switch to fulvestrant
68	The patient is well with no signs of disease	Maintenance therapy with fulvestrant

The initial treatment included breast-conserving surgery, adjuvant radiotherapy (50 Gy to the left breast with a boost of 16 Gy), and the combination of tamoxifen (20 mg per day) and goserelin (3.6 mg subcutaneously once a month). However, four years and two months later, the patient presented with a local recurrence in the right supraclavicular node and a possible mediastinal node, the latter was suspected on imaging but not confirmed with tissue biopsy. She received radiotherapy to the internal mammary nodes and both supraclavicular node stations. Additionally, her hormonal therapy was switched to the aromatase inhibitor exemestane (25 mg daily), and goserelin was continued.

**Figure 1:**
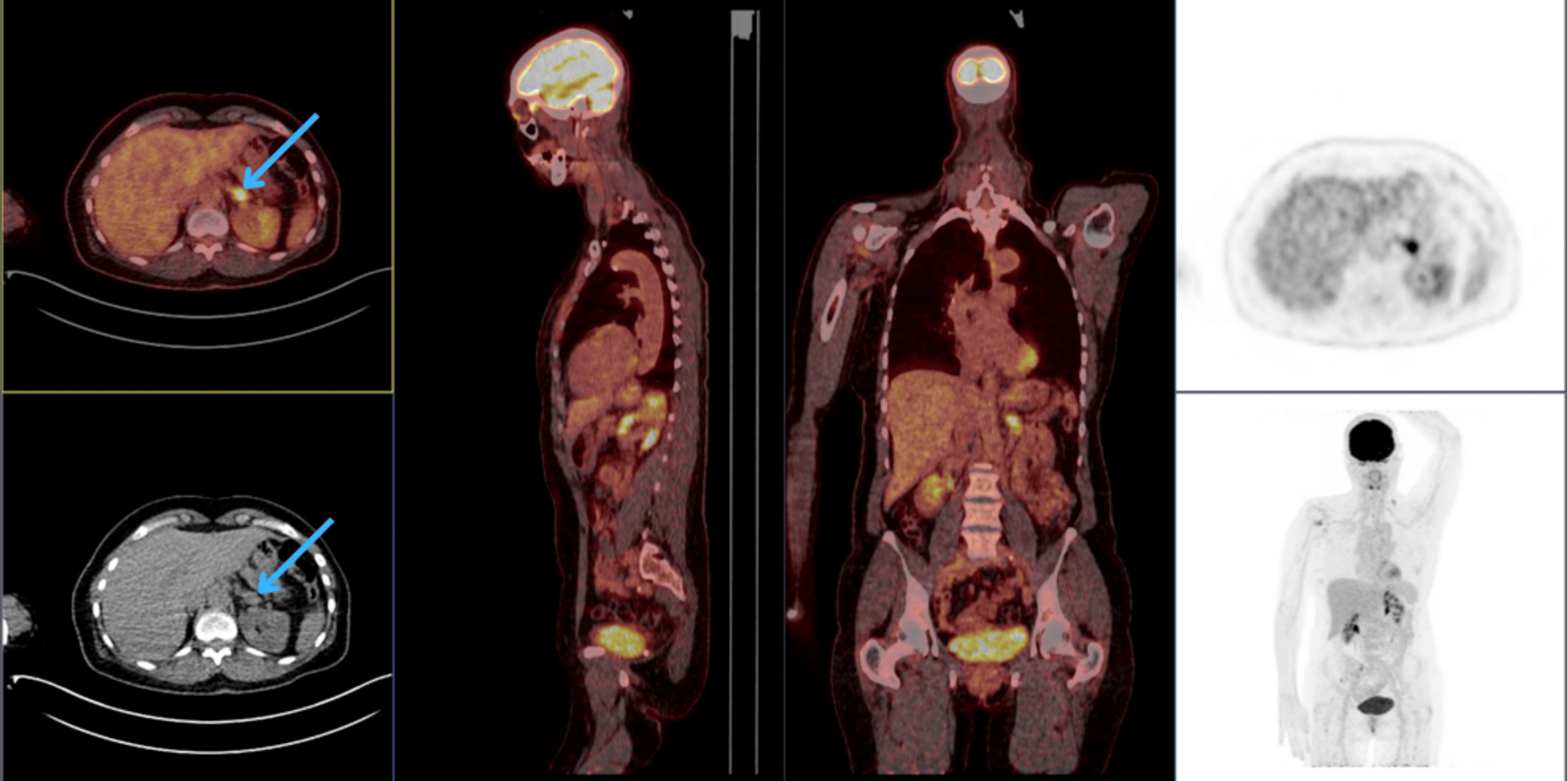
The PET-CT scan showing left adrenal metastasis (blue arrows) PET-CT: Positron emission tomography-computed tomography

**Figure 2:**
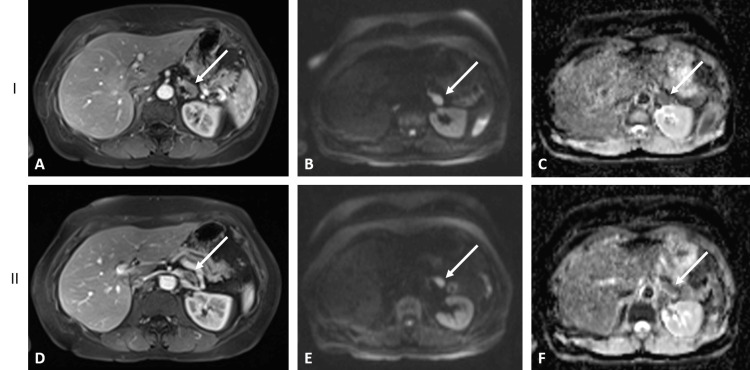
The MRI showing the adrenal metastases (arrows) marked as I and II A and D: Axial T1 fat saturation (FS) after contrast; B and E: Diffusion weighted (B1000); C and F: Apparent diffusion coefficient (ADC)

Since the patient remained premenopausal seven years after the initial diagnosis, both ovaries were removed laparoscopically, allowing for the continuation of exemestane alone. Eight years and seven months later, she experienced a recurrence in the contralateral right axilla. The affected node was excised, with pathology confirming a poorly differentiated (grade 3) tumor, ER-positive, PR-negative, and HER2-negative on IHC and FISH. As adjuvant therapy, she received four cycles of adriamycin and cyclophosphamide, followed by four cycles of docetaxel. Post-chemotherapy, she underwent radiotherapy, and her hormonal therapy was switched to letrozole (2.5 mg). Ten years and eight months after the initial diagnosis, the patient developed marked fatigue, prompting a thorough diagnostic workup. The positron emission tomography-computed tomography PET-CT scan revealed a solitary metastasis in the left adrenal gland (Figure 1). A subsequent MRI provided detailed confirmation of the left adrenal lesion's morphology with a total diameter of 36 mm, further supporting the diagnosis of metastatic involvement (Figure 2).

**Figure 3:**
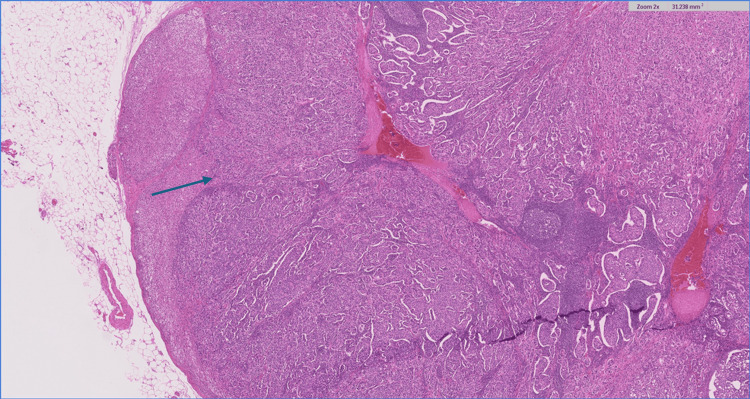
Histological section of the adrenal gland revealing extensive, multifocal intra-adrenal metastasis involving all layers of the adrenal cortex as well as the adrenal medulla (blue arrow); H&E 2x H&E: Hematoxylin and eosin stain

**Figure 4:**
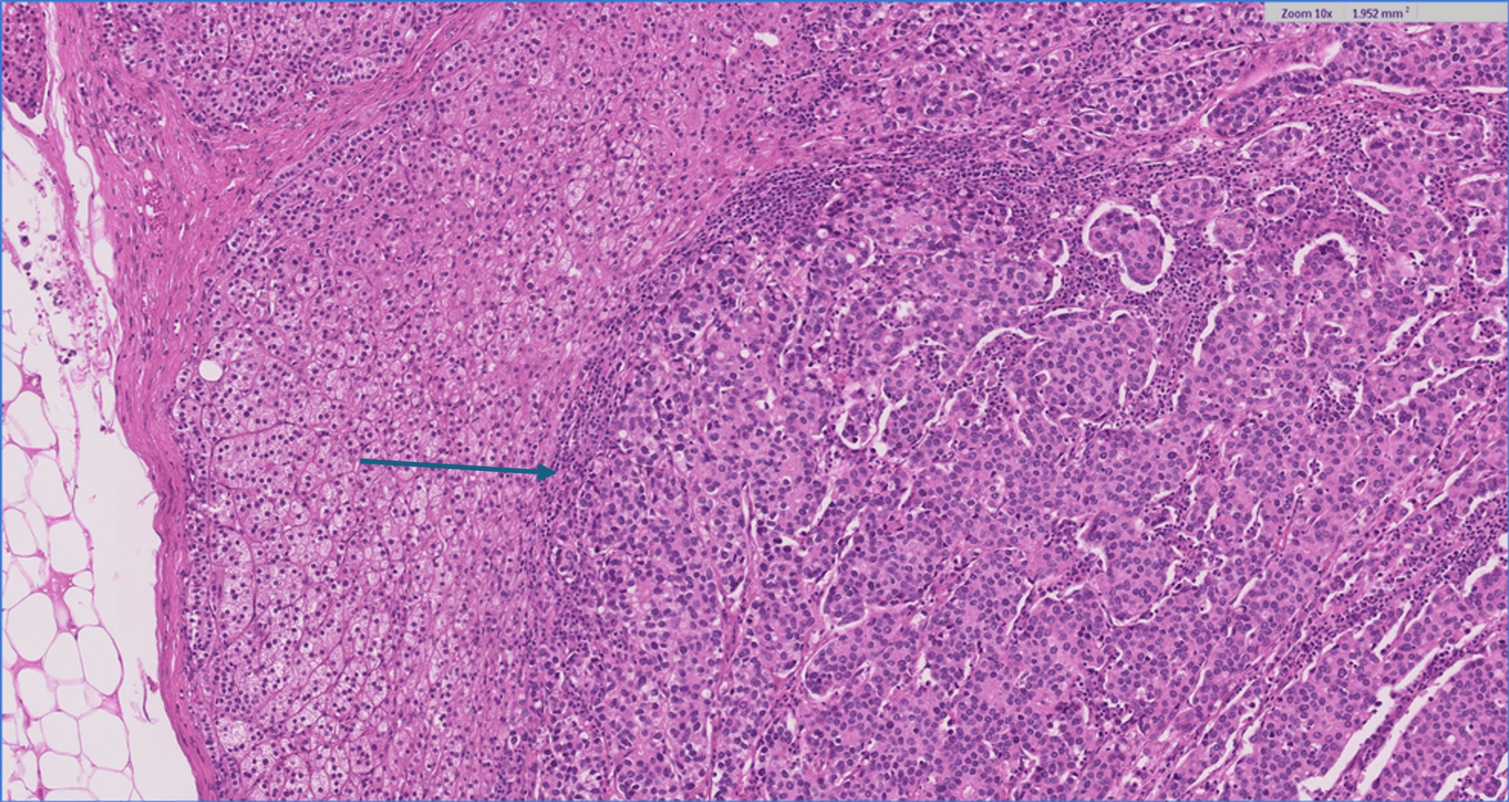
Histological section of the adrenal gland; H&E 10x. The tumor cells are arranged in solid nests or cords with infiltrative growth patterns (blue arrow). H&E: Hematoxylin and eosin stain

Blood analysis including a thyroid function test (thyroid stimulating hormone (TSH), triiodothyronine (T3), and thyroxine (T4)) and cancer antigen (CA) 15-3 were all within normal limits. After a multidisciplinary oncology meeting, an adrenalectomy was advised. A laparoscopic left adrenalectomy was performed, with pathology confirming a 36-mm multifocal intra-adrenal breast cancer. A histological section of the adrenal gland revealed extensive, multifocal intra-adrenal metastasis involving all layers of the adrenal cortex as well as the adrenal medulla. The tumor cells were arranged in solid nests or cords with infiltrative growth patterns (Figures 3-4).

**Figure 5:**
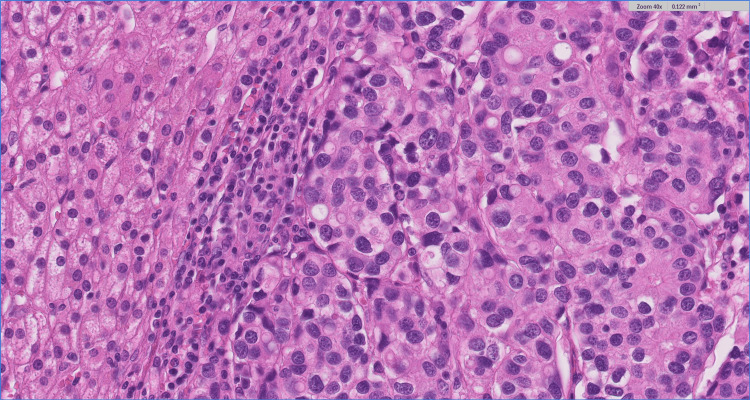
Histological section of the adrenal gland; H&E 40x. The tumor shows varying sizes of oval cells with eosinophilic cytoplasm and prominent small nucleoli. H&E: Hematoxylin and eosin stain

**Figure 6:**
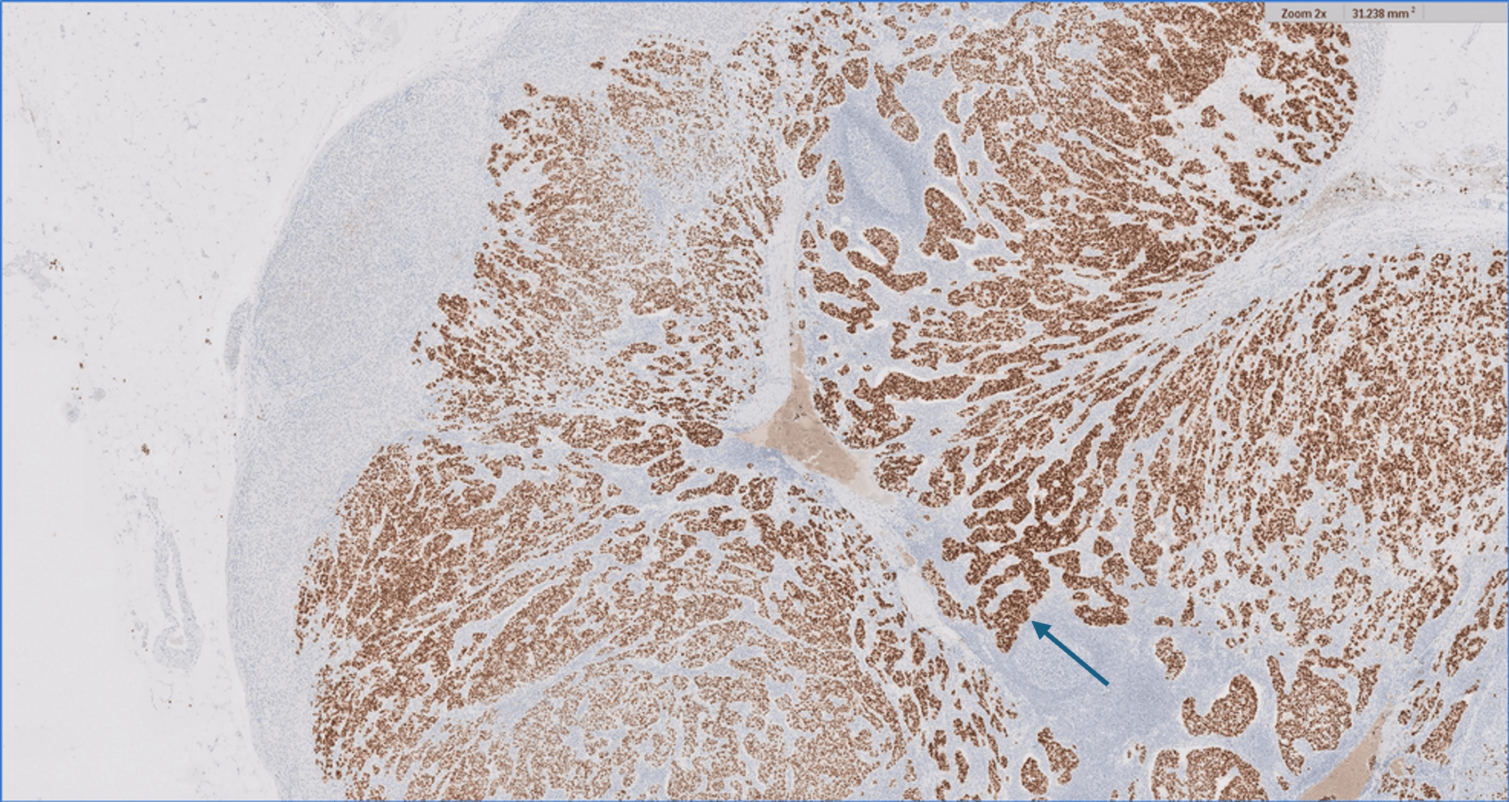
lmmunohistochemical staining with predictive factors (blue arrow; 2x). The tumor cells show positivity for ER (81% to 90%, intense staining). ER: Estrogen receptor

**Figure 7:**
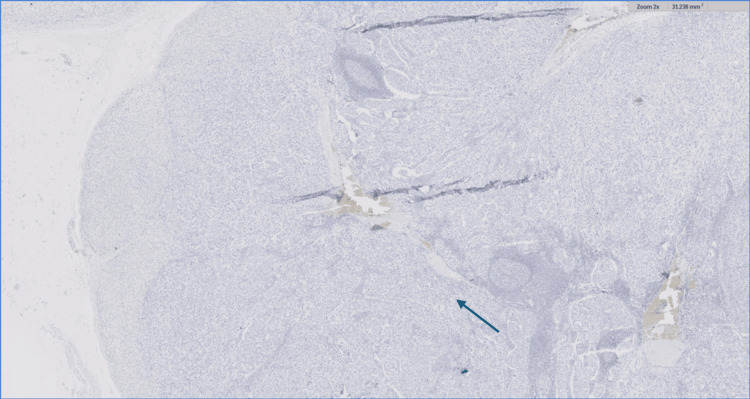
Immunohistochemical staining with the predictive factors (blue arrow; 2x). The PR has a negative (<1%) staining pattern. PR: Progesterone receptor

**Figure 8:**
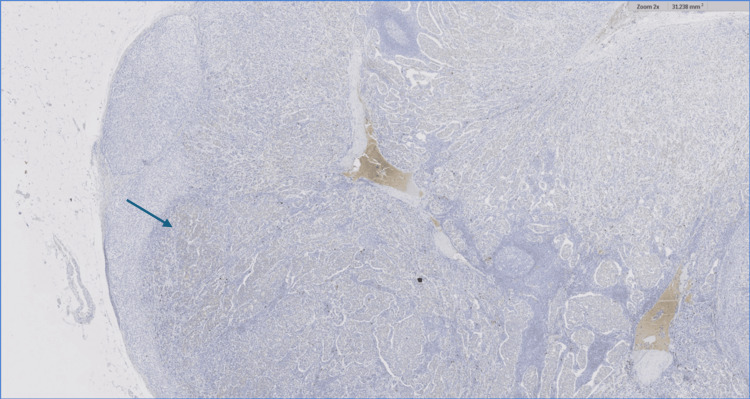
Immunohistochemical staining with predictive factors (2x). The HER2 receptor demonstrates +2 positive staining within tumor cells (blue arrow). HER2: Human epidermal growth factor receptor 2

**Figure 9:**
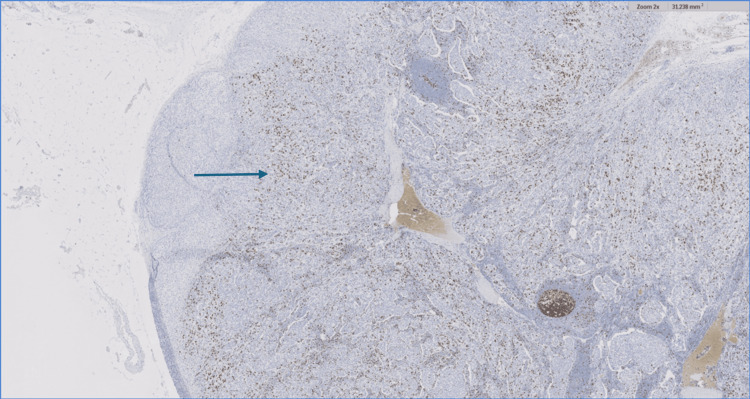
Immunohistochemical staining with predictive factors (2x). The KI67 shows a high proliferation index (blue arrow).

The IHC staining with the predictive factors (2x), showed that the tumor cells were positive for ER (81% to 90%), negative for PR (<1%), positive for HER 2, and the KI67 showed a high proliferation index (Figures 6-9). 

Genetic testing for genes associated with hereditary breast and ovarian cancer (HBOC), including ATM, BARD1, BRCA1, BRCA2, BRIP1, CDH1, CHEK2, MLH1, MSH2, MSH6, PALB2, PTEN, RAD51C, RAD51D, and TP53, did not reveal any pathogenic mutations. Following the adrenalectomy, the patient received fulvestrant (250 mg) monthly. At present, more than 20 years after the initial diagnosis, the patient who is now 68 years old, is still in good health with no signs of disease.

## Discussion

Breast cancer is one of the oldest documented cancers. The first description was probably made by Imhotep, an Egyptian physician, architect, and astrologer, in 3000 BCE. Imhotep was deified as the god of healing [16]. A copy of this text can be found in an Egyptian medical papyrus, the so-called 'Edwin Smith Papyrus,' dated to 1600 BCE [16]. It was documented as a bulging breast tumor without the mention of metastatic spread. More detailed descriptions of local breast cancer can be found in the ancient Greek literature 'Diseases of Women,' a collection of medical works associated with Hippocrates and written 500 to 400 BCE [17]. Probably the earliest description of metastatic breast cancer is by Avicenna (Ibn Sina) (980-1037 AD) in the 'Canon of Medicine' [18]. He described in detail breast cancer and its spread, recognizing the aggressive and invasive nature of metastatic cancer [18].

It was only in 1829 that the concept of metastasis was introduced in the medical world by Récamier, a gynecologist [19]. He used the word metastasis to describe the spread of cancer cells from a primary tumor to distant organs and form secondary tumors. With this, he laid the foundation for understanding the systemic nature of cancer metastasis.

In 1889, Paget noted that the distribution of breast cancer metastases was not random and not due to chance [20], leading to his “seed and soil” theory of metastasis. The basis of this theory is that metastases are driven by cancer cells (“seeds”) growing in a preferred microenvironment (“soil”) of an organ. The organ “selection” for metastasis is based on the implantation of a suitable seed in a suitable soil.

Halsted suggested in 1894 that breast cancer grow “in continuity.” Untreated breast cancer would first spread through regional lymph nodes and then by hematogenous dissemination to the other organs in the body. The idea of linear and centrifugal progression formed the basis for his radical mammectomy and the removal of locoregional lymph nodes [21]. In 1928, Ewing proposed the theory that cancer cells were spread to an organ by the direction of the blood and the lymphatic systems [22]. This is still an interesting and intriguing thought because the blood system is responsible for cellular function and metabolic balance (moving oxygen, nutrients, and waste products like carbon dioxide), and the lymphatic system is critical for immune defense (carrying white blood cells that help fight infections and removing cellular waste and toxins) and fluid balance. Even though on a daily basis, breast cancer patients shed large numbers of tumor cells into the circulation, only a tiny fraction (< 0.1%) of them will give rise to secondary growth; this phenomenon is referred to as “metastatic inefficiency” [8].

Clinically, it is well documented that disseminated tumor cells (DTCs), also called minimal residual disease (MRD), can “store” or survive in the microenvironment of selected organs without developing metastases, a concept referred to as tumor dormancy. In 1934, Willis noted that late metastasis could occur without prior local recurrence, suggesting that the DTCs had invaded secondary sites and remained dormant there [23]. Twenty years later, in 1954, Hadfield expanded on this by proposing that such delayed recurrences could be due to “temporary mitotic arrest” in these dormant cells [23].

In the 1960s, Fisher came up with the concept that breast cancer was a systemic disease and not a local disease [24]. With this theory, he fully recognized the value of Paget’s soil and seed theory. Paget’s initial idea focused on the selectivity in metastasis, while Fisher elaborated on this and focused on how tumor cells interact with and adapt to the microenvironments. The fundamental idea that breast cancer had already spread at the moment of diagnosis changed the basis of breast cancer treatment. It led to the introduction of systemic therapy for the treatment and prevention of these micrometastases, together with the introduction of breast-conservative surgery.

In 1994 Hellman developed the spectrum theory based on Fisher’s systemic view of cancer metastasis [25]. The spectrum theory suggests that cancer metastasis is not simply a binary process (all or nothing) but exists along a continuum, with distinct stages of spread and behavior. One key concept in this continuum is oligometastasis [26]. Oligometastasis is an intermediate state where patients present with a limited number of metastatic sites (typically fewer than five) and the extent of the disease. It suggests that these metastases are less aggressive, might remain more localized, and could therefore be effectively treated with targeted interventions like surgery or radiotherapy. The goal of treating patients with oligometastasis is to prolong survival and the duration of cancer control [27]. It challenges the concept that systemic spread necessarily leads to widespread progression and that treatment is palliation.

Currently, this traditional one-way model of metastasis is challenged by the bidirectional tumor movement of DTCs or circulating tumor cells (CTCs) [12,28-32]. This concept is based on the idea that cancer cells not only spread from the primary tumor to distant sites but also potentially return to the primary tumor. The dynamics of cancer metastasis in a bidirectional movement emphasize the complexity and persistence of cancer cell dissemination​ [32].

Based on the above, it is worthwhile to operate, radiate, or give systemic therapy with curative intent to patients with solitary metastases. The literature and our case report show that in patients with solitary metastases in the adrenal glands or the condition of oligometastatic disease, adrenalectomy can provide improved survival with a good quality of life. Laparoscopic adrenalectomy is a safe method in patients with metastases localized to the adrenal gland with adherence to oncological principles. Open adrenalectomy is indicated in patients with suspicion of infiltration to surrounding structures and metastases in regional lymph nodes. The keystone of good oncological management is that all patients are discussed by a multidisciplinary team (MDT). A multidisciplinary meeting should be done before any kind of treatment. This provides better care for all patients and, in particular, for patients with rare diseases or recurrences. It is a quality indicator for oncological care.

In breast cancer, the likelihood of recurrence varies considerably between subtypes. Recurrence in patients with a luminal type is rare, compared to HER2-positive or basal-like types, and the time of recurrence is also different (Table 2) [2-4]. Luminal A cancers are more likely to recur after 10 years, with the majority of cases occurring later. This emphasizes the importance of long-term follow-up and extended endocrine therapy. Luminal B cancers tend to recur at an earlier stage, often within the first five to 10 years. Cancers that are HER2-positive have a higher risk of recurrence within the first five years, although this risk decreases significantly after this period. Triple-negative cancers have the highest rate of recurrence within the first five years, with very few recurrences after this period.

**Table 2:** Breast cancer subtypes, survival, and recurrence *Either PR low or/and KI67 high HER2: Human epidermal growth factor receptor 2, PR: Progesterone receptor

Subtypes	Luminal A	Luminal B	HER-2	Basal-like
All breast cancer	60% to 70%	10% to 20 %	13% to 15 %	10% to 15 %
ER	Positive	Positive	Positive/Negative	Negative
PR	High positive	Low positive	Positive/Negative	Negative
HER-2	Negative	Negative	Positive	Negative
KI-67	Low	High*	Mainly high	Mainly high
Five-year relative survival	94.4%	90.7%	84.8%	77.1%
Recurrence				
Local	1.5%	2.9%	7.6%	7.5%
Regional	0.7%	1.5%	3.3%	3.4%

Our case of an oligo-metastasis of the adrenal gland in a luminal is even rarer. Evidence regarding the management is therefore sparse. If adrenal metastasis occurs, it is often in a lobular carcinoma and associated with multiple synchronous metastatic sites [14,15]. A literature search was performed (until July 17th, 2024, PudMed) identifying 20 cases of metachronous adrenal gland oligometastasis in patients with breast cancer (Table 3) [14,33-38]. The average age of breast cancer diagnosis was 55 years (range 30 to 79), with 75% of cases classified as ductal carcinoma. The mean size of adrenal metastases was 41 mm (range 20 mm to 70 mm). Estrogen receptor status was positive in six cases, negative in two cases, and unknown in 13 cases. An adrenalectomy was performed in all cases, with adjuvant hormonal therapy and/or chemotherapy in 17 patients. One patient also received trastuzumab. The average time between the initial breast cancer diagnosis and the development of adrenal metastasis was 54 months (range 24 to 128 months), with an interval of 128 months in our case. Excluding our case would reduce the mean interval to 42 months. This interval is nearly three times the mean in previously published cases. The mean survival time after adrenal metastasis diagnosis was 42 months (including our case). Our case had a survival time after metastasis (STAM) of 112 months, and excluding it would reduce the mean to 38 months. Overall survival was reported in only eight cases, with a mean of 78 months (range 30 to 241 months). Excluding our case, the average overall survival decreased by approximately two years to 55 months. The more than 20-year survival in our case is exceptional.

**Table 3:** Characteristics of metachronous solitary adrenal metastasis in patients with breast cancer G: Grade; ?: Unknown; S: Surgery; HT: Hormonal therapy; CT: Chemotherapy; NED: No evidence of disease; DOD: Death of disease; STAM: Survival time after diagnosis of adrenal metastasis; OS: Overall survival ^a ^Interval is the time between breast cancer diagnosis and adrenal metastasis; ^b^STAM; ^c^Of the 13 patients, nine received hormonal therapy, four also received chemotherapy, and one patient received trastuzumab; *The same patient was operated on for bilateral adrenalectomy. The second operation was conducted 15 months after the first adrenalectomy.

Author	Year	Age (years)	TN	Type	ER	PR	HER2	G	I (months)^a^	Size (mm)	Treatment	Status	STAM (months)^b^	OS (months)
Liu et al. [8]	2010	64	T2N1	Ductal	-	-	+	2	24	54 x 70	S	NED	36	60
Yoshitomi et al. [10]	2012	42	T4bN0	Ductal	+	+	1+	?	48	?	S + HT	NED	28	76
Mizuyama et al. [11]	2013	47	T3N1	Ductal	-	-	3+	?	24	?	S + CT	NED	6	30
Andjelic et al. [12]	2014	58	T2N2	Ductal	+	+	3+	2	36	48 x 49	S + CT	DOD	27	63
Barros et al. [13]	2015	61	T2N1	Ductal	+	?	?	3	60	40 x 70	S	NED	0	60
He et al. [14]	2016	30	?	Ductal	+	+	3+	?	60	25	S^c^	NED	8	33
Illuminati et al. [9]^C^	2021	61	?	Ductal	?	?	?	?	?	40	S^c^	NED	77	?
		68	?	Lobular	?	?	?	?	?	30	S^c^	DOD	28	?
		54	?	Lobular	?	?	?	?	?	20	S^c^	DOD	33	?
		42	?	Ductal	?	?	?	?	?	50	S^c^	NED	72	?
		69	?	Ductal	?	?	?	?	?	30	S^c^	NED	64	?
		76	?	Lobular	?	?	?	?	?	60	S^c^	NED	23	?
		36	?	Ductal	?	?	?	?	?	70	S^c^	NED	89	?
		28	?	Lobular	?	?	?	?	?	40	S^c^	DOD	32	?
		47	?	Ductal	?	?	?	?	?	30	S^c^	NED	108	?
		79	?	Ductal	?	?	?	?	?	20	S^c^	NED	41	?
		77	?	Ductal	?	?	?	?	?	30	S^c^	NED	40	?
		70*	?	Ductal	?	?	?	?	?	50	S^c^	NED	15	?
		70*	?	Ductal	?	?	?	?	?	20	S^c^	NED	2	?
Sun et al. [15]	2022	48	T2N1	Mucinous	+	+	+	?	60	35	S	NED	0	60
Present	2024	48	T1N0	Ductal	+	+	-	3	128	36	S + HT	NED	113	241

Metachronous isolated adrenal metastases are rare for lobular and even rarer for ductal breast cancers. The difference in incidence is unlikely due to the different growth patterns. Lobular will often display more favorable histopathological characteristics in comparison to ductal; the latter is more frequently a high nuclear-grade lesion. The amount of distant recurrence is similar, yet they display different metastatic behavior. Bone is the most common site in both subtypes. Lobular is known to cause more skin metastases and gastrointestinal metastases, whereas ductal metastases to the lung occur more often. The metastatic spread in ductal is mainly in bone (35.5% to 37.7%), lung and pleura (17.6% to 24.4%), liver (10.9% to 11.4%), and central nervous system (5.3% to 6.1%) [39]. Despite all the differences, the overall outcome is similar for lobular and ductal [40].

Historically, adrenal metastases in patients with a history of primary malignancy range between 10% and 27% [41]. During life, adrenal metastases rarely give signs of adrenal insufficiency. There are only symptoms of adrenal insufficiency in the case of large involvement or bleeding that destroys great parts of the adrenal gland.

Diagnostic management

Adrenal metastasis can be detected by ultrasound, CT, MRI, and PET. A lesion can be confused with primary adrenal tumors. A CT scan and MRI help differentiate metastasis from a primary adrenal tumor [14]. The positive predictive value of PET in correctly identifying adrenal metastatic disease is 83% [42]. Due to the increased use of PET-CT in oncology, incidentalomas will be detected in increasing numbers. Adrenal metastases rarely give signs of adrenal insufficiency. There are only symptoms of adrenal insufficiency in the case of large involvement or bleeding that destroys great parts of the adrenal gland. In the past, the majority of the adrenal metastases were only detected during autopsy. The final diagnosis is made by either a biopsy or an adrenalectomy. The 20% false-positive rate for PET-positive adrenalectomies performed for metastatic disease should warrant its inclusion in preoperative counseling to the patient and interaction with the treating oncologist [42].

Therapeutic intervention

The prognosis of metastatic breast cancer is generally unfavorable. For this reason, many clinicians choose palliative care instead of curative therapy. The survival rate differs tremendously between patients with oligometastasis and polymetastases. In recent years, more and more clinicians have opted for an aggressive approach if there is oligometastasis [43]. Breast cancer patients with oligometastasis treated by aggressive multidisciplinary treatments can have a relapse-free rate of 42% at 20 years [43].

There are currently no guidelines for the management of patients with solitary adrenal metastasis. Based on our data from the literature on cases of metachronous solitary adrenal metastases in patients with breast cancer (Table 3) and data from selected patients with and without concomitant extra-adrenal metastases, long-term survival after adrenalectomy is possible [14,33-38,44]. It is crucial that such patients are discussed in a multidisciplinary team meeting.

A laparoscopic adrenalectomy is preferred over open surgery due to the reduced morbidity and an overall equal outcome [14]. Surgery should ideally be combined with systemic therapy (chemotherapy, targeted therapies, and antihormonal therapies).

Our decision to start fulvestrant after the detection of the solitary left adrenal metastasis was based on the knowledge we had at that time. Systemic therapies for breast cancer, however, are evolving continuously based on findings from randomized controlled trials (RCTs). Regular updates to guidelines are available on the European Society for Medical Oncology (ESMO) and National Comprehensive Cancer Network (NCCN) websites [45-47]. The management of hormone receptor-positive and HER2-negative metastatic breast cancer with isolated adrenal metastasis requires a multidisciplinary oncology approach, where personalized local and systematic treatment options are thoroughly discussed. The emphasis should be placed on biomarker-guided strategies to optimize clinical outcomes.

The preferred first-line treatment is a cyclin-dependent kinase 4/6 (CDK4/6) inhibitor combined with endocrine therapy, such as an aromatase inhibitor or fulvestrant. Second-line therapy for patients with a PIK3CA mutation includes alpelisib with fulvestrant or inavolisib with palbociclib and fulvestrant [45-47]. For patients with PIK3CA or AKT1 mutations, or PTEN alterations, capivasertib combined with fulvestrant can be considered [45-47]. In cases with ESR1 mutations, elacestrant is an available option [45-47]. For germline BRCA-positive patients, treatment may also include poly-ADP ribose polymerase (PARP) inhibitors such as olaparib or talazoparib [45-47].

The current case and literature review demonstrate the need for a lifelong (inter)national cancer registry for rare recurrences in frequent cancers. This registry would offer detailed data on the frequency of these uncommon events and their corresponding treatments, helping to guide the development of consistent treatment strategies. Ideally, treatment recommendations regarding rare recurrences or rare cases should be discussed by a (inter)national multidisciplinary team instead of a local multidisciplinary team, especially in a small country like Belgium. This way, the formulation of a 'uniform' treatment approach is feasible, leading undoubtedly to better survival of patients.

## Conclusions

A solitary adrenal metastasis from breast cancer is extremely rare. Due to the rareness, there is no standard management. If a solitary metastasis is discovered, it should be discussed at a multidisciplinary team meeting, and an en-bloc resection of the metastasis with systemic therapy is the first choice when feasible. The present case reveals also that there is a need for a lifelong (inter)national cancer registry for rare recurrences or cases. This would be a reference source and allow (inter)national multidisciplinary team discussion resulting in improved survival and quality of life for the patients.
